# Multicenter Pivotal Clinical Trial of Urine Malaria Test for Rapid Diagnosis of Plasmodium falciparum Malaria

**DOI:** 10.1128/JCM.01431-16

**Published:** 2016-12-28

**Authors:** Wellington A. Oyibo, Nnenna Ezeigwe, Godwin Ntadom, Oladipo O. Oladosu, Kaitlin Rainwater-Loveth, Wendy O'Meara, Evaezi Okpokoro, William Brieger

**Affiliations:** a ANDI Centre of Excellence for Malaria Diagnosis, International Malaria Microscopy Training and Rapid Diagnostic Test Quality Assurance Programme, and WHO/TDR/FIND Malaria Specimen Bank Site, College of Medicine, University of Lagos, Lagos, Nigeria; b National Malaria Elimination Program, Federal Ministry of Health, Abuja, Nigeria; c Johns Hopkins University School of Public Health, Baltimore, Maryland, USA; d Duke University School of Medicine, Durham, North Carolina, USA; e Institute of Human Virology Nigeria, Abuja, Nigeria; UNC Health Care System

**Keywords:** health care provider, malaria, noninvasive malaria test, Plasmodium falciparum, point-of-care diagnosis, primary healthcare setting, rapid diagnostic test (RDT), urine malaria test (UMT)

## Abstract

The need to expand malaria diagnosis capabilities alongside policy requirements for mandatory testing before treatment motivates exploration of noninvasive rapid diagnostic tests (RDTs). We report the outcome of the first cross-sectional, single-blind clinical performance evaluation of a urine malaria test (UMT) for diagnosis of Plasmodium falciparum malaria in febrile patients. Matched urine and finger-prick blood samples from participants ≥2 years of age with fever (axillary temperature of ≥37.5°C) or with a history of fever in the preceding 48 h were tested with UMT and microscopy (as the gold standard). BinaxNOW (Pf and Pan versions) blood RDTs were done to assess relative performance. Urinalysis and rheumatoid factor (RF) tests were conducted to evaluate possible interference. Diagnostic performance characteristics were computed at 95% confidence intervals (CIs). Of 1,800 participants screened, 1,691 were enrolled; of these 566 (34%) were febrile, and 1,125 (66%) were afebrile. Among enrolled participants, 341 (20%) tested positive by microscopy, 419 (25%) were positive by UMT, 676 (40%) were positive by BinaxNOW Pf, and 368 (22%) were positive by BinaxNow Pan. UMT sensitivity among febrile patients (for whom the test was indicated) was 85%, and specificity was 84%. Among febrile children ≤5 years of age, UMT sensitivity was 93%, and specificity was 83%. The area under the receiver-operator characteristic curve (AUC) of UMT (0.84) was not significantly different from that of BinaxNOW Pf (0.86) or of BinaxNOW Pan (0.87), indicating that the tests do not differ in overall performance. Gender, seasons, and RF did not impact UMT performance. Leukocytes, hematuria, and urobilinogen concentrations in urine were associated with lower UMT specificities. UMT performance was comparable to that of the BinaxNOW Pf/Pan tests, making UMT a promising tool to expand malaria testing in public and private health care settings where there are challenges to blood-based malaria diagnosis testing.

## INTRODUCTION

The current WHO recommendation for parasitological confirmation of suspected malaria cases prior to treatment is a paradigm shift from entrenched presumptive diagnostic practices. Annually, there are 214 million estimated cases of malaria globally (1). Despite increasing parasitological diagnosis capabilities in countries of endemicity, universal access to testing in cases of fever suspected of being induced by malaria remains a major challenge, particularly in community and private health care settings. This is due in part to limited access to quality-assured microscopy, laboratory infrastructure requirements, blood safety concerns, and regulatory/policy requirements that forbid nonlaboratory professionals from undertaking invasive diagnostic procedures.

Among 18 nationally representative surveys conducted in Africa from 2013 to 2015, the median proportions of febrile children receiving malaria testing was 53% in public health facilities, 36% in the formal private sector, and 6% in the informal private sector, with only 12.6% of febrile children being tested in Nigeria (2, 3). Therefore, there is substantial opportunity for malaria diagnoses, particularly in the private health care sector, where most fevers are managed (3). Consequently, if the goal of universal access to diagnosis is to be achieved and national and international diagnosis targets are to be met, novel technologies will play a significant role. Progress in diagnostic coverage requires wider access to safe, effective, low-cost, and easy-to-use tests at primary-level points of care, including community health workers, private clinics, pharmacies, medicine shops, and front-line government health facilities. Several new tests are under development, such as technologies that target parasite antigen (e.g., fluorescent RDTs) and hemozoin, a point-of-care test for spectroscopy, and serology, which is currently commercially available as ELISA tests in blood bank screening in developed countries (4–6). Among these tests, the urine malaria test (UMT) developed by Fyodor Biotechnologies, Inc. (Baltimore, MD, USA), is the only nonblood malaria test that has undergone a full-scale premarket evaluation trial.

Previous attempts to use urine and saliva for malaria diagnosis in blood-based RDTs produced poor performance (7–13). The UMT is a simple, immuno-chromatographic dipstick that utilizes recombinant monoclonal antibody to detect highly repetitive cognate polyhistidine-rich protein 2 (HRP-2) and fragments thereof shed in the urine of febrile patients (D. J. Sullivan and P. Scholl, U.S. patent application 20090117602). A pilot field study that tested the UMT in 203 febrile patients showed a sensitivity of 83.75%, specificity of 83.48%, positive predictive value (PPV) of 77.91%, and negative predictive value (NPV) of 88.07% (14), indicating the viability of an accurate urine-based malaria test. The study enrolled predominantly children (73.8%), and the outcome also indicated the need for a larger study population to broaden the range of patients, including those without objective fever at presentation.

Here, we report the outcome of a pivotal multicenter clinical performance evaluation of the UMT in Lagos State, Nigeria.

## RESULTS

### Demographics and clinical characteristics of patients.

Of 1,800 participants screened, 1,691 (94%) were enrolled per eligibility criteria, with a mean age (± standard deviation [SD]) of 18.6 years (SD of 15.9; range, 2 to 80), of whom 970 were female (57%) and 721 were male (43%). Among these 1,691 enrollees, 566 (34%) participants were febrile (≥37.5°C), and 1,125 (66%) were afebrile but reported a history of fever in the previous 48 h (Table 1). Headache, body pain, and chills were the most commonly reported symptoms in both groups. Normal urine ketone levels, pH, and specific gravity were observed in 80%, 85%, and 75% of participants, respectively.

**TABLE 1:** Demographic characteristics and clinical history of the study population

Parameter	No. of patients (%)		
	Overall (*n* = 1,691)	Febrile (*n* = 566)	Afebrile (*n* = 1,125)
Gender			
Female	970 (57)	276 (49)	694 (62)
Male	721 (43)	290 (51)	431 (38)
Age (yr)			
2–5	416 (25)	202 (36)	214 (19)
6–11	390 (23)	175 (31)	215 (19)
12–17	195 (12)	81 (14)	114 (10)
18–20	61 (4)	20 (4)	41 (4)
21–39	427 (25)	71 (13)	356 (32)
40–54	153 (9)	13 (2)	140 (12)
55+	49 (3)	4 (1)	45 (4)
Symptoms			
Chills	1,135 (67)	442 (78)	693 (62)
Body pain	1,159 (69)	387 (68)	772 (69)
Headache	1,364 (81)	473 (84)	891 (69)
Vomiting	257 (15)	129 (23)	128 (11)
Stomach ache	185 (11)	128 (23)	57 (5)
Weakness	68 (4)	56 (10)	12 (1)
Anorexia	186 (11)	86 (15)	100 (9)
Cough	189 (11)	117 (21)	72 (6)
Catarrh	170 (10)	107 (19)	63 (6)
Bitter taste	31 (2)	25 (4)	6 (1)
Dizziness	46 (3)	35 (6)	11 (1)
Other	83 (5)	25 (4)	58 (5)
Study site			
Agura	365 (22)	75 (13)	290 (26)
Bayekun	218 (13)	88 (16)	130 (12)
Ijede	522 (31)	206 (36)	316 (28)
Imota	439 (26)	164 (29)	275 (24)
Oreta	48 (3)	6 (1)	42 (4)
Shomolu	99 (6)	27 (5)	72 (6)

^a^ The mean age ± SD for each group was as follows: overall, 18.6 ± 15.9 years (range, 2.0 to 80 years); febrile, 11.6 ± 11.0 years (range, 2.0 to 70 years); afebrile, 22.1 ± 16.7 years (range, 2.0 to 80 years).

### Malaria prevalence.

Malaria prevalence varied among the overall, febrile, and afebrile participant populations. Overall, 341 (20%) of 1,691 enrollees were microscopy-positive for malaria (Table 2). The UMT detected malaria in 419 (25%) of participants, while BinaxNOW tests that detect the HRP-2 antigen of Plasmodium falciparum (BinaxNOW Pf) and aldolase, a pan-malaria antigen found in all Plasmodium species (BinaxNOW Pan), detected malaria in 676 (40%) and 368 (22%) of participants, respectively. The highest prevalence was among febrile participants. Among 566 febrile participants, 204 (36%) were positive by microscopy, 231 (41%) were positive by UMT, 224 (40%) were positive by BinaxNOW Pan, and 317 (56%) were positive by BinaxNOW Pf. Similarly, febrile participants had higher parasite densities (PDs) than afebrile participants (Table 2) (2). Three participants with monospecies Plasmodium malariae infection by microscopy were negative by UMT and BinaxNOW Pf, while two of them were positive by BinaxNOW Pan. These monospecies samples were excluded in the analyses.

**TABLE 2:** Malaria microscopy, UMT, blood-based malaria RDT (BinaxNow Pf/Pan), and urinalysis results of the study population

Parameter	No. of patients (%)^*b*^		
	Overall (*n* = 1,691)	Febrile (*n* = 566)	Afebrile (*n* = 1,125)
Positive malaria diagnostic tests			
Microscopy	341 (20.2)	204 (36)	137 (12.2)
UMT	419 (25)	231 (41)	188 (17)
BinaxNOW Pf	676 (40)	315 (56)	361 (32)
BinaxNOW Pan	368 (22)	224 (40)	144 (13)
Parasite prevalence			
Detectable asexual stage parasites	338 (20)	203 (36)	155 (14)
Density (no. of parasites)^*a*^			
1–199	2 (1)	1 (0.5)	1 (1)
200–499	16 (5)	5 (2)	11 (7)
500–999	20 (6)	8 (4)	12 (8)
1,000–4 999	70 (21)	34 (17)	36 (23)
5,000–9 999	44 (13)	26 (13)	18 (12)
10,000–49 999	109 (32)	75 (37)	34 (22)
50,000+	77 (23)	54 (27)	23 (15)
Urinalysis			
Leukocytes >15/µl	189 (11)	67 (12)	182 (16)
Presence of nitrites (mg/dl)	22 (1)	7 (1)	15 (1)
Urobilinogen of ≥1 mg/dl	159 (9)	67 (12)	92 (8)
Protein of >100 mg/dl	73 (4)	42 (7)	31 (3)
Ketone of >5 mg/dl	344 (20)	176 (31)	168 (15)
Bilirubin of ≥1 mg/dl	159 (9)	71 (13)	88 (8)
Glucose (mg/dl)	21 (1)	5 (1)	16 (1)
Blood (erythrocytes/µl)	175 (10)	77 (14)	98 (9)
pH			
<7	1,443 (85)	493 (87)	950 (84)
7	114 (7)	45 (8)	69 (6)
>7	130 (8)	27 (5)	103 (9)
Specific gravity			
<1.010	549 (32)	158 (28)	391 (35)
1.011–1.020	725 (43)	264 (47)	461 (41)
>1.021	414 (24)	144 (25)	270 (24)

a Asexual parasite stage only.

b Percentages in subcategories of parasite density were calculated as number of patients in the density subcategory/total number of patients with detectable asexual parasites.

### Performance of the UMT. (i) Febrile participants.

UMT sensitivity among the 566 febrile participants was 85% (95% confidence interval [CI], 79, 89), and specificity was 84% (95% CI, 80, 88) (Table 3). Among febrile children of ≤5 years of age, UMT sensitivity was 93% (95% CI, 80, 98), and specificity was 83% (95% CI, 75, 89). Compared to UMT, BinaxNOW Pf sensitivity was high (99% [95% CI, 97, 100]), while specificity was low (69% [95% CI, 64, 74]) (both *P* < 0.001). The sensitivity and specificity of BinaxNOW Pan did not differ significantly from those of UMT, while PPVs and NPVs did not differ significantly between UMT and either of the BinaxNOW tests. The negative likelihood ratio (NLR) for UMT differed from that of BinaxNOW Pf and was closer to that of BinaxNow Pan, while the positive likelihood ratio (PLR) for UMT was also closer to that of BinaxNow Pan than to that of BinaxNow Pf (Table 3).

**TABLE 3:** Performance of urine malaria test and blood-based RDTs in overall study participants and febrile and afebrile subjects at clinical presentation

Group and parameter^*a*^	Test performance^*b*^			*P* value^*c*^	
	UMT	BinaxNOW Pf	BinaxNOW Pan	UMT vs BinaxNOW Pf	UMT vs BinaxNOW Pan
All participants (*n* = 1,691)					
Sensitivity (% [95% CI])	79 (75, 84)	98 (96, 99)	80 (76, 84)	<0.001	0.824
Specificity (% [95% CI])	89 (87, 91)	75 (72, 77)	93 (92, 94)	<0.001	<0.001
PPV (% [95% CI])	65 (60, 69)	50 (46, 53)	74 (70, 79)	1.00	0.988
NPV (% [95% CI])	94 (93, 96)	99 (99, 100)	95 (94, 96)	0.932	1.00
NLR	0.23 (0.23, 0.24)	0.02 (0.02, 0.03)	0.21 (0.21, 0.22)		
PLR	7.2 (7.2, 7.3)	3.9 (3.9, 3.9)	11.5 (11.3, 11.8)		
Febrile participants (*n* = 566)					
Sensitivity (% [95% CI])	85 (79, 89)	99 (97, 100)	86 (80, 90)	<0.001	0.883
Specificity (% [95% CI])	84 (80, 88)	69 (64, 74)	86 (83, 90)	<0.001	0.176
PPV (% [95% CI])	75 (69, 80)	64 (59, 69)	78 (72, 83)	1.00	0.981
NPV (% [95% CI])	91 (87, 94)	99 (97, 100)	92 (88, 94)	0.693	0.808
NLR	0.18 (0.17, 0.19)	0.01 (0.01, 0.04)	0.16 (0.16, 0.17)		
PLR	5.3 (5.1, 5.5)	3.2 (3.1, 3.2)	6.3 (6.1, 6.6)		
Afebrile participants (*n* = 1,125)					
Sensitivity (% [95% CI])	72 (63, 79)	97 (93, 99)	72 (64, 80)	<0.001	1.00
Specificity (% [95% CI])	91 (89, 93)	77 (74, 80)	95 (94, 97)	<0.001	<0.001
PPV (% [95% CI])	52 (45, 59)	37 (32, 42)	69 (61, 76)	1.00	0.981
NPV (% [95% CI])	96 (94, 97)	99 (99, 100)	96 (95, 97)	0.938	1.00
NLR	0.31 (0.30, 0.32)	0.04 (0.02, 0.06)	0.29 (0.28, 0.30)		
PLR	7.9 (7.7, 8.1)	4.2 (4.2, 4.2)	15.9 (15.1, 16.6)		

a PPV, positive predictive value; NPV, negative predictive value; NLR, negative likelihood ratio; PLR, positive likelihood ratio.

b BinaxNOW Pf contains HRP-2, a *P*. falciparum-specific antigen, and BinaxNOW Pan contains aldolase, an antigen found in all species of Plasmodium. P. falciparum is the most dominant species in the trial area.

c *P* values comparing the sensitivities and specificities of the Fyodor UMT and the BinaxNOW tests were estimated using McNemar's test. *P* values comparing the positive and negative predictive values were calculated using the weighted generalized score statistic.

### (ii) Afebrile patients.

Of 1,125 afebrile participants, UMT sensitivity was 72%, and while it did not significantly differ from that of BinaxNOW Pan (72%) (*P* = 1.00), sensitivity was significantly lower than that of BinaxNOW Pf (97%) (*P* = 0.001) (Table 3). The specificities of UMT and BinaxNOW Pan among afebrile participants were 91% (95% CI, 89, 93) and 95% (94, 97), respectively, and significantly higher than the specificity of BinaxNOW Pf (77% [95% CI, 74, 80]) (*P* < 0.001). The likelihood ratios also varied among the tests.

### (iii) Overall study population (febrile and afebrile).

Overall UMT sensitivity and specificity for all participants (febrile and afebrile combined) were 79% (95% CI, 75, 84) and 89% (96% CI, 87, 91), respectively. UMT sensitivity did not differ from that of BinaxNOW Pan (80%) but was significantly lower than that of BinaxNow Pf (98% [95% CI: 96, 98]) (*P* < 0.001) (Table 3). Specificity was higher than that of the BinaxNOW Pf test (75% [95% CI, 72, 77]) and lower than that of BinaxNOW Pan (93% [95% CI, 92, 94]) (both, *P* < 0.001). The PPV and NPV were ≤75% and ≥94%, respectively, for all tests and did not differ significantly from each other. The BinaxNOW Pf also differed from UMT and BinaxNOW Pan with respect to NLR and PLR values in the general study population (Table 3).

### Overall diagnostic value of UMT and BinaxNOW tests.

**FIG 1:**
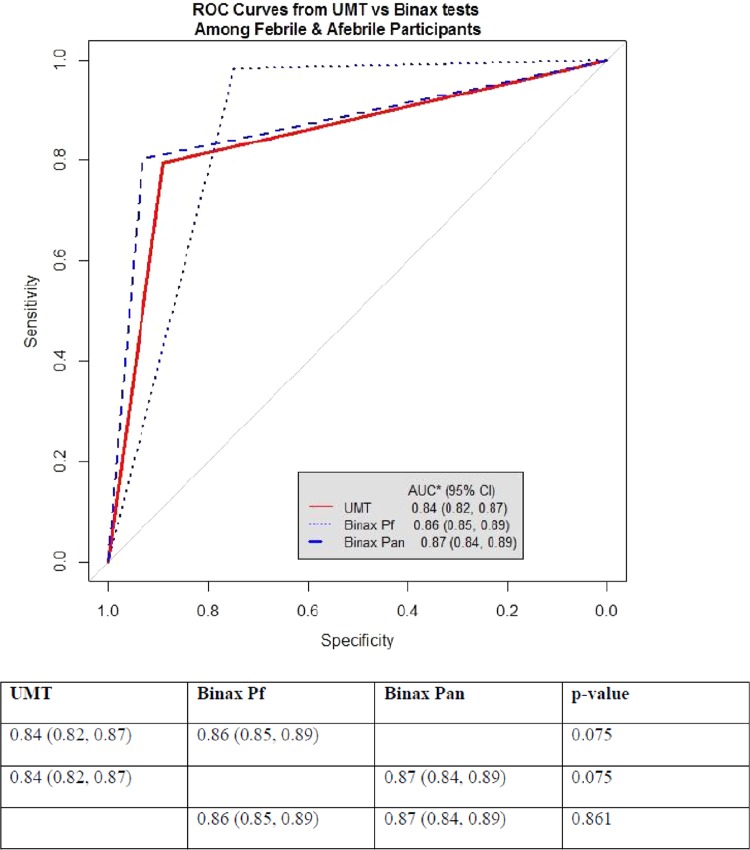
Pooled ROC curves of the study participants (combined febrile and afebrile) tested with the urine malaria test and BinaxNOW Pf and BinaxNOW Pan. AUC, area under the curve. *P* values comparing the area under the receiver-operator characteristic (ROC) curves were computed using DeLong's test for two ROC curves.

The values of the area under the receiver-operator characteristic (ROC) curves (AUCs) for UMT (*P* = 0.212), BinaxNOW Pf (*P* = 0.054), and BinaxNOW Pan (*P* = 0.358) did not differ between febrile and afebrile participants (all, *P* > 0.05) (Table 4). A pooled AUC calculated for each diagnostic test (Fig. 1) showed that the UMT (0.84 [95% CI, 0.82, 0.87]) was not significantly different from the BinaxNOW Pf (0.86 [95% CI, 0.85, 0.89]) or BinaxNOW Pan (0.87 [95% CI, 0.84, 0.89]) test, suggesting that the tests do not differ in overall performance.

**TABLE 4:** Area under the ROC curve for all tests stratified by presence of fever

Test	AUC (95% CI)		*P* value^*a*^
	Febrile	Afebrile	
UMT	0.84 (0.81, 0.88)	0.81 (0.78, 0.84)	0.212
BinaxNOW Pf	0.84 (0.81, 0.87)	0.87 (0.85, 0.89)	0.054
BinaxNOW Pan	0.86 (0.83, 0.89)	0.84 (0.81, 0.87)	0.358

a *P* values comparing the area under the receiver-operator characteristic (ROC) curves were computed using DeLong's test for two ROC curves.

### Other performance characteristics.

Graded PDs by microscopy compared with UMT performance showed that increased PD was associated with increased probability of fever (*P* < 0.001). UMT, BinaxNOW Pf, and BinaxNOW Pan sensitivities increased with higher PDs among all patients, as well as with stratified subpopulations of febrile and afebrile patients (Table 5). While BinaxNOW Pf sensitivity was highest across PDs and stratified subpopulations, the UMT and BinaxNOW Pan specificities were higher than the specificity of BinaxNOW Pf.

**TABLE 5:** Sensitivity and specificity of UMT, BinaxNOW Pf, and BinaxNOW Pan tests by parasite density level for febrile, afebrile, and all study participants

Group and test	Test performance by parasite density^*a*^				*P* value by density group^*b*^	
	Specificity (% [CI]) at 0	Sensitivity (% [CI])				
		1–999	1,000–4,999	5,000+	1–999	1,000–4,999
Total study population						
UMT	89 (87, 91)	58 (41, 74)	69 (56, 79)	86 (81, 90)	<0.001	0.002
BinaxNOW Pf	75 (72, 77)	95 (82, 99)	96 (88, 99)	100 (98, 100)	0.055	0.042
BinaxNOW Pan	93 (92, 94)	42 (26, 59)	70 (58, 80)	90 (85, 93)	<0.001	<0.001
Febrile group						
UMT	84 (80, 88)	71 (42, 92)	76 (59, 89)	88 (81, 92)	0.105	0.107
BinaxNOW Pf	69 (64, 74)	93 (66, 100)	97 (85, 100)	NA	0.083	0.181
BinaxNOW Pan	86 (83, 90)	57 (29, 82)	79 (62, 91)	NA	0.004	0.143
Afebrile group						
UMT	91 (89, 93)	50 (29, 71)	61 (43, 77)	82 (71, 90)	<0.001	0.020
BinaxNOW Pf	77 (74, 80)	96 (79, 100)	94 (81, 99)	99 (93, 100)	0.055	0.253
BinaxNOW Pan	95 (94, 97)	33 (16, 55)	61 (43, 77)	90 (81, 96)	<0.001	0.001

a Parasite density (number of parasites/microliter of blood) was determined by microscopy. NA, not available.

b *P* values are based on a comparison with the results from the 5,000+ density group using Fisher's exact test.

The sensitivities of all tests decreased with increasing age and increased with higher PDs. In contrast, specificities of all tests were significantly higher in adults than in younger participants in the overall study population (all, *P* < 0.001). The PPVs of UMT did not differ between age categories, but while NPVs of children and adolescents were significantly lower than those of adults (*P* < 0.001), all values were ≥90% in each category. Upon stratification by fever, the majority of the performance characteristics of the three tests did not differ significantly between the oldest and youngest participants.

Gender and season (dry and rainy) did not affect UMT performance. Urinalysis revealed that a higher number of leukocytes (>15) was associated with a significantly higher specificity (*P* = 0.010), while hematuria was associated with significantly lower specificity (*P* = 0.001). UMT specificity was lower among participants with urobilinogen concentrations of ≥1 mg/dl than in those with levels of <1 mg/dl (*P* < 0.001). Specific gravity, which indicates urine concentration, was not associated with UMT performance. All of the 15 rheumatoid factor-positive (RF^+^) participants tested negative for malaria and were negative by UMT.

### PCR correction of microscopy results.

The original microscopy results showed that 365 individuals were infected with Plasmodium parasites: 362 (99%) were infected with P. falciparum, and 3 (1%) were infected with P. malariae. A further analysis of these samples by PCR showed that seven study samples originally found to harbor P. falciparum by microscopy were negative by PCR. Additionally, 58 microscopy-negative samples were positive by PCR; of these 56 were infected with P. falciparum, and 2 were infected with P. malariae. The PCR results of one participant sample indicated infection with Plasmodium ovale, but the source of the infection was originally identified by microscopy as P. falciparum.

The six participants infected with non-P. falciparum parasites as determined by PCR were removed from the data set. There were no statistically significant differences between original and PCR-corrected microscopy results of discordant samples by UMT and BinaxNOW Pf/Pan, likely reflecting high-quality microscopy methods and well-trained microscopists (Table 6).

**TABLE 6:** Urine malaria test (UMT) performance characteristics among study participants infected with Plasmodium falciparum relative to original and PCR-corrected microscopy results

Parameter and group^*a*^	UMT performance relative to:		*P* value^*b*^
	Original microscopy	PCR-corrected microscopy	
Sensitivity (% [CI])			
All participants	78 (74, 83)	77 (72, 81)	0.665
Febrile	85 (79, 89)	81 (75, 86)	0.577
Afebrile	72 (64, 79)	72 (66, 79)	1.00
Specificity (% [CI])			
All participants	90 (89, 91)	92 (90, 93)	0.368
Febrile	84 (80, 88)	85 (81, 89)	0.770
Afebrile	92 (90, 93)	94 (92, 95)	0.091
PPV (% [CI])			
All participants	63 (58, 67)	70 (65, 74)	1.00
Febrile	74 (68, 80)	77 (71, 82)	1.00
Afebrile	51 (44, 57)	63 (56, 69)	1.00
NPV (% [CI])			
All participants	95 (94, 96)	94 (93, 95)	0.945
Febrile	90 (87, 93)	88 (84, 91)	0.891
Afebrile	97 (95, 97)	96 (95, 97)	0.941
PLR			
All participants	0.240 (0.237, 0.243)	0.253 (0.249, 0.256)	
Febrile	0.201 (0.195, 0.207)	0.228 (0.221, 0.236)	
Afebrile	0.304 (0.298, 0.311)	0.294 (0.287, 0.302)	
NLR			
All participants	7.87 (7.72, 8.03)	9.25 (9.11, 9.40)	
Febrile	5.29 (5.04, 5.55)	5.49 (5.29, 5.70)	
Afebrile	8.70 (8.42, 8.99)	11.2 (10.9, 11.5)	

a PPV, positive predictive value; NPV, negative predictive value; PLR, positive likelihood ratio; NLR, negative likelihood ratio.

b *P* values were estimated by the chi-square statistic for sensitivity and specificity and by the weighted generalized score method for predictive values.

The original microscopy results indicated that 361 study participants were infected with P. falciparum, while PCR-corrected microscopy results indicated that 410 participants had P. falciparum malaria, and the urine malaria test indicated that 451 participants were infected with P. falciparum. UMT sensitivities, specificities, and predictive values changed by less than 3% regardless of whether all study participants, febrile participants, or afebrile participants were assessed, by between 0 and 7% for BinaxNOW Pf, and by between 0 and 11% for BinaxNOW Pan. Overall, the largest changes occurred among the positive predictive values (PPVs), but this was expected as a mathematical relationship between prevalence and PPV value is known, whereby increasing prevalence always produces an increased PPV. Likelihood ratios also did not demonstrate clinically meaningful changes.

In contrast to microscopy results, those for sensitivity, specificity, and negative predictive values changed by less than 3% regardless of which participant population, total, febrile, or afebrile, was assessed. The largest changes occurred among the positive predictive values (PPVs), with differences between the original and PCR-corrected microscopy results ranging from 3% to 12%. However, this was expected as a mathematical relationship between prevalence and the PPV value is known, whereby increasing prevalence always produces an increased PPV. Likelihood ratios also did not demonstrate clinically meaningful changes.

## DISCUSSION

The UMT is the first noninvasive malaria test clinically evaluated on a large scale at the population and community levels. Given its sensitivity and specificity, a disease prevalence of 20% in the study population, and a PLR of 7.2, the posterior probability of disease in a patient who tests positive by the UMT is 59%. Given the NLR of 0.23, the posterior probability of disease in a patient who tests negative by UMT is 4%. This indicates that the UMT could aid in the clinical management of suspected malaria cases. For example, upon receiving a negative UMT result for a suspected malaria case, the clinician now knows that this patient's probability of having malaria parasites detectable by microscopy is unlikely, i.e., only 4%. In contrast, a positive UMT result would indicate that the probability of detecting malaria parasites in this patient by microscopy is 59%.

The performance of UMT was higher among febrile participants (who are the intended use population) than among afebrile individuals. High rates of false positives and lower specificities reported among HRP-2-based blood RDTs corroborate our findings (15–20) and are worrisome in spite of their high sensitivity. Broader clinical utility of a rapid malaria test should be based on its overall diagnostic value using the AUC of the receiver-operator characteristics. Our data support a role for the UMT as a diagnostic or screening tool at the control phase in countries with high to medium malaria transmission rates. However, test performance may be lower in low-transmission regions or preelimination countries, as reported for blood RDTs (21–25). Nevertheless, field evaluation studies in different transmission settings would provide performance data on UMT to demonstrate its utility.

It should be noted that P. falciparum parasitemia and antigenemia do not correlate directly due to sequestration, while there are no data on the correlation between parasitemia and antigenuria. Therefore, UMT performance metrics must be established *de novo* and not on the basis of antigenuria reported by RDTs.

The availability of the blood-based RDTs has greatly increased malaria testing particularly in the public sector, where about 53% of patients with malaria-like symptoms are tested. In the formal and informal private sector health care settings, only 36% and 6%, respectively, of patients are tested (26). The UMT could potentially expand malaria testing in private health care settings, particularly in hard-to-reach locations or health care facilities where blood draw is difficult or impractical for microscopy, and advance the current global effort toward universal diagnosis in cases of fever suspected of being malaria.

Viral hemorrhagic fevers (such as Ebola viral disease [EVD]) are major global health concerns where the UMT could be invaluable. The Ebola viral proteins connected with infection are present in blood and other body fluids at the time of fever and onset of symptoms (27, 28) but are rarely found in patient urine (29, 30). Thus, in outbreak situations, the UMT could potentially offer a safer and rapid diagnostic tool to rule out malaria as the cause of fever, particularly in rural communities with severely limited laboratory facilities.

### Conclusion.

Programmatically, the UMT could expand access to prompt parasitological confirmation of malaria in public and private health care settings, particularly in areas or settings where invasive blood testing is impractical or restricted.

## MATERIALS AND METHODS

### Study area.

The study was conducted in six health care facilities (HFs) across Ikorodu (rural/suburban) and Somolu (urban) local government areas (LGAs) of Lagos State, southwest Nigeria. Malaria is hypo-mesoendemic in Lagos State, with peak transmission during the rainy season (April to September). Ikorodu LGA and Somolu LGA have estimated populations of 535,619 and 402,763, respectively.

### Study sites.

The HFs in Ikorodu LGA were the following: Imota Primary Health Center (PHC), Imota; Agura PHC, Gberigbe; Ijede Health Center, Ijede; Bayekun PHC, Bayeku; Annex of St. Kizito Clinic/PHC, Oreta; and General Hospital, Somolu, Somolu LGA, Lagos State, Nigeria.

### Study design and enrollment criteria.

This cross-sectional, single-blind, multicenter clinical performance study screened 1,800 participants of both genders (≥2 years of age) who presented with fever (axillary temperature, ≥37.5°C) or history of fever in the past 48 h. Fifteen blood smear-negative rheumatoid factor positive (RF^+^) individuals were enrolled and tested to determine potential cross-reactivity with UMT, as RF is known to elicit proteinuria and cross-reactivity with HRP-2-based RDTs (31–33). Individuals who reported use of antimalarial medications within the last 2 weeks and those with severe disease were excluded. The study was conducted from July 2013 to February 2014 and covered the rainy and dry seasons.

### Power and sample size justification.

Simulations for the joint confidence region of sensitivity and specificity showed that at least 1,300 study participants with fever or history of fever were required, powered at 80% at a 0.05 limit of indifference (Fisher's exact conditional test for two proportions and Walter's normal approximation), assuming that 40% of subjects would have positive blood smears, based on the “Nigeria Malaria Indicator Survey 2010” (3).

### Sample collection and handling.

Upon enrollment, a case report form was completed for each participant. Matched finger-prick blood and urine samples were collected for malaria microscopy, RDT, UMT, and urinalysis. Blood spots were prepared from finger-prick blood samples on Whatman filter paper (GE Healthcare Life Science, United Kingdom). RF testing was done on a cohort of microscopy-negative patients.

### UMT technology and procedure.

The UMT technology relies on the fact that in clinical malaria, febrile patients shed elevated levels of proteins, including P. falciparum proteins, in urine (9), and the test detects HRP-2 shed in the urine of individuals infected with malaria. The UMT (Fyodor Biotechnologies, Inc., Baltimore, MD, USA) was performed per the manufacturer's instructions. Briefly, a test strip was dipped into a labeled 2-ml test tube containing 200 μl of urine and incubated for 10 min. The strip was removed and incubated for 15 min at room temperature, after which the result was read. No clinical decision regarding patient care was made based on UMT results.

### BinaxNOW (Pf/Pan) malaria RDT, urinalysis, rheumatoid arthritis test (RF test), and dry blood spot procedure.

The BinaxNOW (Pf/Pan) blood-based malaria RDT (Alere, Inc., Waltham, MA, USA) is a qualitative immunochromatographic assay that detects HRP-2 antigen found in P. falciparum (BinaxNOW Pf), and aldolase, a pan-malaria antigen found in all Plasmodium species (BinaxNOW Pan). The test was performed per the manufacturer's instructions and was used as a comparator RDT. Multianalyte urinalysis reagent strips (Rapid Labs, Essex, United Kingdom) were used to measure glucose, bilirubin, specific gravity, ketones, blood, pH, protein, urobilinogen, nitrites, serum creatinine, and leukocytes. A direct slide test was used to detect RF (Arlington Scientific, Inc., USA). All tests were performed per the manufacturer's instruction.

### Malaria microscopy.

Thick and thin malaria blood films (MBFs) were prepared on the same slide in HFs and transported to the ANDI Center of Excellence for Malaria Diagnosis at the University of Lagos for microscopy. Two WHO-accredited malaria microscopists blinded to UMT and blood RDT (BinaxNOW Pf/Pan) results read the MBFs independently. Asexual parasites were counted relative to 200 to 1,000 leukocytes in thick films. Computed parasite density (PD) was reported as the number of parasites/microliter of blood using the mean PD of the two microscopists if discordance was ≤20%. Discordance in PDs of >20% between the two microscopists required reading by a third WHO level 1 microscopist, and the PD was recomputed incorporating this microscopist ‘s findings.

### PCR.

PCR corrections of microscopy on discordant test results were performed within the scope of another study in collaboration with Stanford University using primers targeting the Pfr364 repetitive element (34) for P. falciparum detection and the pan-Plasmodium 18S rRNA gene (35, 36).

### Ethical consideration/study quality assurance.

Written informed consent/assent was obtained from each participant and/or parent/guardian. This study was conducted in accordance with good clinical practice and guidance from the Nigerian Health Research Ethics Code. Patients with positive malaria RDT results were treated per national malaria diagnosis and treatment guidelines. The study was approved by the Ethics Committee of the College of Medicine, University of Lagos, Nigeria. The Lagos State Ministry of Health approved the use of HFs. A Data and Safety Monitoring Committee reviewed study and data management processes. Independent study audits were undertaken by the Nigerian National Agency for Food, Drug Administration and Control, and by Regulatory and Quality Solutions, LLC, Murrysville, PA. Study protocol and reporting conformed to the standards for reporting of diagnostic accuracy. The study has been registered at ClinicalTrials.gov under registration number NCT01921413.

### Statistical analyses.

Microscopy was the gold standard (reference test) for sensitivity and specificity calculations. Parasite density (number of parasites per microliter of blood) was computed for all positive MBFs. Exact binomial confidence intervals were calculated for sensitivity, specificity, and positive and negative predictive values (PPVs and NPVs, respectively). McNemar's test was used to compare sensitivities and specificities between diagnostic tests. The weighted generalized score method was used to compare predictive values (37). The chi-square test was used to compare the number of participants testing positive by each rapid test across categorical variables. Fisher's exact test was used to compare the number of participants testing positive by each rapid test across dichotomous variables when appropriate. A Cochran-Mantel-Haenszel test was used to compare the odds of detecting malaria parasites by microscopy between 24- and 48-h fever durations among participants who tested positive by each rapid test. All analyses were conducted in R, version 3.0.1 (http://www.R-project.org/), using the packages epicalc, pROC, and ROCR.
